# How a Spanish Group of Millennial Generation Perceives the Commercial Novel Smoothies?

**DOI:** 10.3390/foods9091213

**Published:** 2020-09-01

**Authors:** Marina Cano-Lamadrid, Karolina Tkacz, Igor Piotr Turkiewicz, Jesús Clemente-Villalba, Lucía Sánchez-Rodríguez, Leontina Lipan, Elena García-García, Ángel A. Carbonell-Barrachina, Aneta Wojdyło

**Affiliations:** 1 Research Group “Food Quality and Safety”, Department of Agro-Food Technology, Escuela Politécnica Superior de Orihuela (EPSO), Universidad Miguel Hernández de Elche (UMH), Ctra. Beniel, km 3.2, 03312 Orihuela, Alicante, Spain; marina.cano.umh@gmail.com (M.C.-L.); jesus.clemente@hotmail.com (J.C.-V.); lucia.sanchez@goumh.umh.es (L.S.-R.); leontina.lipan@yahoo.com (L.L.); egarcia@umh.es (E.G.-G.); angel.carbonell@umh.es (Á.A.C.-B.); 2 Vegetable and Plant Nutraceutical Technology, Department of Fruit, Wrocław University of Environmental and Life Sciences, 37 Chełmońskiego Street, 51-630 Wrocław, Poland; karolina.tkacz@upwr.edu.pl (K.T.); igor.turkiewicz@upwr.edu.pl (I.P.T.)

**Keywords:** Napping^®^, descriptive sensory analysis, JAR, PLS, drivers of liking, penalty analysis

## Abstract

The World Health Organization (WHO) and the Food and Agriculture Organization of the United Nations (FAO) constantly emphasize the importance of increasing fruit and vegetable consumption; these natural products help in the prevention of major diseases. Smoothies are a simple and convenient way of doing so; thus, their demand is constantly growing and their market is becoming important for the food industry. Therefore, the objective of this research was to determine Millennial consumer opinion towards novel fruit- and vegetable-smoothies available on the retail market. Napping^®^, descriptive sensory analysis, and consumer studies were conducted. Napping^®^ results group samples into four clusters of smoothies; the main grouping factor was the type of fruit and the percentage of vegetables. Penalty analysis showed that smoothies need improvement mainly dealing with sweetness, bitterness, and vegetable flavors. Millennial consumers formed a homogeneous sensory group in which the overall liking was negatively correlated with the level of sweetness, and earthy, carrot, beetroot, and pear flavors. The key liking drivers were sourness and notes of mango, banana, and peach flavors. This research is a new insight into the perception of smoothies, provides comprehensive knowledge for the food industry, and can guide the design of new healthy smoothies.

## 1. Introduction

Fruits and vegetables intake is highly recommended and necessary in a healthy diet due to their important nutrients and bioactive constituents (phenolic compounds, vitamin C, anthocyanins, among others) that was demonstrated in several studies to diminish the risk of chronic non-communicable diseases [1]. The Mediterranean diet, which has been widely studied regarding its effect on the prevention of diabetes and low-density lipoprotein oxidation, includes fruits and vegetables (tomato, broccoli, carrot, etc.) with high content of phytochemicals (carotenoids, phenolic compounds, vitamins, foliates, minerals, etc.) [2]. Nutritional recommendations point to the consumption of fruit and vegetables as part of a balanced diet, although with a reduction in the consumption of juices and smoothies due to the possible excessive supply of energy from sugars. In addition, these products have a moderate or high glycemic index and, as a consequence, can cause a rapid increase in glucose and insulin levels. However, smoothies are rich in soluble fiber (pectin, mucus) and insoluble fiber (cellulose and lignin), which delays the absorption of monosaccharides into the bloodstream and thus effectively regulates glucose-insulin homeostasis [3].

The World Health Organization (WHO) is still alerting that 71% of all deaths worldwide are caused by non-communicable diseases (NCDs), and every year 15 million people die prematurely, i.e., between the ages of 30 and 69 years, from NCD. Unfortunately, there is still a lack of public health actions about fruits and vegetables consumption (e.g., health education and health program promotion); this is true for both developing countries but also for developed countries due to the current lifestyle [4,5]. The recommended fruit and vegetables intake is 400 g per day. To encourage, to maintain and/or to increase their intake, the food industry comes with an alternative to raw fruits and vegetable intake, developing new products, easy-to-eat, and with longer shelf-life than regular fruits, such as smoothies [1]. In 2018, although consumption average of raw fruits and vegetables per person in Spain was 412 g (247 g and 155 g for fruits and vegetables, respectively) [6], part of the Spanish population are below the recommended intake (no available information about the percentage of people who are consuming less than the recommended level).

The word “smoothie” comes from the English term “smooth” (tender, creamy), and defines a creamy non-alcoholic drink with a thick texture similar to that of milkshakes. This beverage includes only natural ingredients such as puree fruit with fruit juice, and possibly dairy products or/and crushed ice cubes [5,7]. Their preparation is based on the use of the entire fruit, which is processed from pulp to puree, with only the seeds and peel being removed. To develop different flavors and to obtain the appropriate texture of the final product, the juice from different fruits is used, and it is important to highlight that they are prepared without adding preservatives, stabilizers, or chemical correctors of pH and acidity [8]. This type of product is the easiest form to eat fruits and vegetables and to increase its consumption between consumers with the current lifestyle [5].

The smoothies market can be divided into several segments, attending to different criteria (e.g., product type, distribution channel, and geographical location). Based on product type, the market is segmented into fruit-based, vegetal-based, dairy-based, and others. The fruit-based smoothies segment accounts for the largest market and is expected to be the fastest growing segment in the healthy beverage market. The global smoothies market were worth $12.1 billion in 2020 and is projected to reach a compound annual growth rate (CAGR) of 8% over the next five years, to reach a value of $17 billion in 2025 [9]. North America dominates the smoothie market, while the Asia-Pacific region is expected to be the fastest growing area. It is worth mentioning that, although an increase in smoothies’ consumption exists in those areas, people’s health have worsened during recent years, basically increasing overweight problems. On the other hand, despite the health-promoting potential of smoothies, in Spain, it is still a sub-segment. However, consumer interest is growing, driven by health and wellness trends. Therefore, it is essential to test their sensory quality to improve their consumption [10].

Nowadays, several research studies about the food habits of the Millennial generation (those born between 1980 and 2000) have been carried out and now there is information about their needs and demands. The literature shows the uniqueness of Millennials and this is why current food sensory studies are focusing on this specific population [11]. On the other hand, although sensory analysis has been previously applied to smoothies [12,13,14,15,16,17], there is a lack of information about the opinion and preference of commercial smoothies by consumers, including Millennial ones (generation Y) on smoothies; this is relevant because this generation constitutes the largest group consuming healthy minimally processed foods. In a previous consumer study, Polish consumers indicated that smoothies containing cranberry, black currant, dog rose, and bilberry purees were more acceptable than other fruits [18].

Descriptive sensory analysis allows defining the food product in the context of its appearance, aroma, taste and texture, and also gives the opportunity to link these descriptive data with consumer preferences. Several studies about smoothies used sensory descriptive analysis to characterize them. For instance, a recent study of different cultivars of pomegranate indicated that the smoothies prepared with *Mollar de Elche* pomegranate juice were characterized by having high intensity of pear odor/aroma and consistency, and the *Wonderful* smoothies were characterized by lower consistency and more intense pomegranate aroma and sourness [17]. Therefore, the aim of this study was to determine a Spanish group of Millennial consumers preference towards novel fruit and vegetable smoothies available on the retail market. Napping^®^, descriptive sensory analysis, and consumer studies were conducted. The research will provide a new insight into the perception of smoothie products and comprehensive knowledge for the food industry and can guide the design of new foods, including functional and healthy fruit-based products.

## 2. Materials and Methods

### 2.1. Experimental Design

The research consisted of two parts:

- In the first part of the study (session 1), a Napping^®^ test was conducted focusing on the smell and taste of the 14 fruit- and vegetable-based smoothies; 100 consumers participated in this session. The objective of this part was to narrow down the selection of samples for second part of the study.
- In the second part of the study, a descriptive sensory evaluation by a trained panel and a consumer study (session 2) was conducted using only randomly selected smoothies by researchers, representing the clusters shown in the napping study. The consumers panelist for consumer study was the same people as the Napping test. The objective of this part was to know the consumer‘s liking drivers of types of smoothies (clusters).

With this design, the number of smoothie products in the first stage was 14, while in the second one, 4 samples were used. Two weeks passed between Session 1 and Session 2, data collection and statistic were needed before Session 2 for the selection of the appropriated sample of each detected cluster.

The sensory tests were carried out in a special tasting room with individual booths (controlled temperature of 21 ± 1 °C and combined natural/artificial light), and ballot charts were used to collect panelists’ evaluations. The samples were presented according to a randomized block design to avoid biases. Samples were served chilled at 4 °C into odor-free, disposable 90 mL covered plastic cups, at room temperature and were coded using 3-digit numbers. In each vessel, 40 mL was added in all sensory tests carried out. Trained panelists and consumers were instructed to drink the served portion of the sample for doing descriptive sensory analysis, Napping test, and consumer study, respectively. Unsalted crackers and water were provided to panelists to clean their palates between samples.

All panelists (descriptive test) and consumers (Napping and affective tests) gave their informed consent for inclusion before they participated in the study. Universidad Miguel Hernández de Elche automatically exempts “general taste tests”, including descriptive sensory tests from needing ethical approval, based on European Union guidelines. However, the study was conducted in accordance with the Declaration of Helsinki, and the protocol was approved by the Ethics Committee of the Escuela Politécnica Superior de Orihuela, Universidad Miguel Hernández de Elche.

### 2.2. Smoothie Samples

Fruit- and vegetable-based smoothies were purchased on the retail market (supermarkets and grocery stores) in the province of Alicante (Spain) in 2018. The products were stored in their original unit packets under refrigerated conditions (as indicated on packaging: 4 °C) until analysis. Each smoothie came from 1 batch, and the general sample destined for analysis consisted of 4 primary samples. The compositions of smoothies declared by the producers are given in Table 1.

### 2.3. Consumer Information

A Spanish group of Millennial consumers (*n* = 100) were recruited among the students of Pharmacy Degree at the Miguel Hernández University (UMH). The ages ranged from 20 to 35 years old (born between 1985 and 1999), being 56% females and 44% males. First, they participated in the napping test (session 1) and later in a consumer study with the 4 selected smoothies (session 2).

### 2.4. Napping^®^ Technique

Napping^®^ is a relatively new and rapid sensory profiling technique based on positioning products on a paper sheet (from French “nappe”—tablecloth) to collect sensory distance perceives among products. Data are processed using multivariate techniques such as Generalized Procrustes Analysis (GPA) and Multiple Factor Analysis (MFA). Finally, a Napping^®^ test creates sample clusters according to their specific organoleptic attributes and the own definitions given by the panelists.

The napping test was performed focusing on the flavor of the 14 fruit- and vegetable-based smoothies in session 1. Explanations on how to perform the test was provided to participants at the beginning. Each consumer was provided with a 60 cm × 40 cm blank paper, which was approximately A2 size (the napping sheet), a pen and a tray with the 14 samples. The sample order on the individual trays was randomized to avoid the effect of order of presentation and the carryover effect of a preceding sample over a series [19], even though the napping methodology allows and requires subjects to go back and forth among samples. Samples were coded with 3-digit random numbers.

Consumers were instructed to evaluate samples according to similarities or dissimilarities in flavor attributes by placing similar samples close to each other and more dissimilar samples further apart on the napping sheet. They were asked to complete the task using their own criteria and they were told that there were no right or wrong answers. After they had reached a final configuration, consumers wrote down appropriate descriptors for flavor notes of the samples, which were moved around the napping sheet, when needed. This procedure is known as ultra-flash profiling and is commonly used to add a descriptive dimension to a napping task [20]. When all the samples had been placed on the paper, they replaced samples with an X and noted the sample codes and the smoothie descriptors next to the X. Re-tastings and spitting out the smoothies were allowed. Water and crackers were used as palate cleansers.

From the 4 clusters generated in this part of the study, 1 representative model smoothie of each cluster was selected for the second part of this research (session 2).

### 2.5. Descriptive Sensory Analysis

Eight trained panelists (aged 25 to 55 years; 4 females and 4 males) with more than 600 h of training in sensory testing from the department of Agro-Food Technology (UMH) participated in this study. The panel was selected and trained following the ISO standard 8586-1 (1993), and it is specialized in descriptive sensory evaluation of fruit products [17,21,22,23]. For the present study, the panel worked during 2 orientation sessions (90 min for each one) discussing the main organoleptic characteristics of commercial smoothies. The lexicon used was based on the previous ones developed by other authors [17,22,24,25,26,27] (Table 2). Lexicons were adapted for smoothies during the orientation sessions. Samples were assessed using sensory attributes of basic tastes, flavor notes and texture and somatic sensations (*n* = 22), and the order process was (i) flavor notes, (ii) basic tastes, and (iii) texture and somatic sensations. References were chosen and prepared according to previous publications using similar attributes [17,22,24,25,26,27], and then provided to panelists. The scale to be used was range from 0 (no intensity) to 10 (extremely high intensity) with 0.5 increments.

### 2.6. Consumer Studies

The consumer study was carried out in the tasting rooms of UMH. Each consumer tasted all 4 selected samples in a single session (session 2). Consumers were asked about their overall liking on a nine-point hedonic scale (1 =dislike extremely, 5 = neither like nor dislike, and 9 = like extremely) followed by questions about the appearance, flavor, and texture attributes. Additionally, a nine-point scale was used for the Just About Right (JAR) questions (1, 2, and 3 mean deficit of each attribute, too low in Penalty Analysis; 4, 5 and 6 mean “Just About Right”, JAR in Penalty Analysis; and, 7, 8 and 9 mean excess, too high in Penalty Analysis) to determine possible improvements of the attributes: color, bitterness, sourness, sweetness, fruity, vegetal, and viscosity. These results provide direction of improving, but it does not provide information on degree of intensity change and ingredients adjustment.

### 2.7. Statistical Analysis

Two consecutive tests were performed: (i) one-way analysis of variance (ANOVA), and (ii) Tukey’s multiple range test. Homogenous groups and the least significant difference (LSD) were determined at a significance level of *p* < 0.05 (95% of confidence level). Data were subjected to ANOVA, after checking the normality and homogeneity of the variance, and later to Tukey’s multiple-range test to compare the means. Overall, liking data (y: dependent variable) versus descriptive sensory analysis (x: independent variables) were used to perform partial least squares regression (PLS). In addition, penalty analysis was conducted to provide extra information about the possible improvements of some samples. All statistical analyses were performed using StatGraphics Plus 5.0 software (The Plains, VA, USA) except Napping, Penalty Analysis, PLS, and dendrogram were prepared using XLSTAT Premium 2016 software (2016, París, Frane).

## 3. Results and Discussion

### 3.1. Results of Napping^®^

Appearance, including the color and texture of fruits-based drinks, is the first quality parameter evaluated by consumers and is a key tool for product selection and, then, acceptance or rejection. However, taste is more strongly correlated with the overall assessment of products and is the most relevant acceptability feature used by consumers to evaluate fruit and vegetable products [1,28]. Therefore, in the napping^®^ test, consumers evaluated only “flavor” attributes of 14 smoothies. The terms and their frequencies of mentions for each sample (Table 3) were added as supplementary variable in the Multiple Factor Analysis (MFA). The terms were, thus, not included in the construction of the MFA factors but projected in the product space [19].

Consumers described the taste and aroma (perception of volatile compounds with the product within the mouth) using a total of 34 different sensory descriptors. Each of the smoothies was described with an average of 310 terms (average of number of terms of each smoothie, see Table 3) which were used and repeated by consumers depending on the product. Nevertheless, the most frequently used terms were fruity, citric, tropical fruit, sweetness, and sourness (from 273 to 382 repetitions for each of these terms for the 100 consumers). The average number of mentions of these attributes per product was above 20. The terms caramel, artificial, persistent, unpleasant, aromatic, unknown, and soft were used with a lower frequency (maximum 36 repetitions of these terms in whole task).

**Figure 1:**
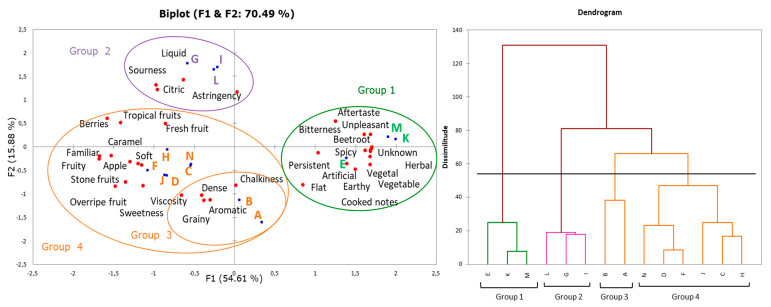
Multiple factor analysis biplot of terms used by a Spanish group of Millennial consumers for commercial smoothies in the Napping^®^ test and dendrogram using Pearson’s correlation based on the unweighted average.

The frequency and number of sensory descriptors for each smoothie were subjected to multiple factor analysis and the outcomes are shown on the MFA biplot (Figure 1). The first two dimensions (F1 and F2) explained 70.49% of the total variance (54.61% and 15.88%, respectively) (*p*-value < 0.01, alpha = 0.05). Additionally, the results were supported by a dendrogram using Pearson’s correlation based on the unweighted average (Figure 1). Millennial consumers created four representative clusters composed of the following smoothies and the most characteristic attributes:

1. Group 1: Smoothies E, K and M were described using the attributes vegetal, vegetable, herbal, earthy, spicy, cooked notes, beetroot, bitterness, and unpleasant, among others. These products had a high content in forms of juices or/and purees of carrot, beet, celery, cucumber, spinach and kale, and small additions of ginger and lemon.
2. Group 2: Smoothies G, I, and L were defined as liquid, sour, citric and astringent. These smoothies contained high percentages of orange, strawberry, raspberry, blueberry, pomegranate, carrot, pumpkin, lemon, pineapple in forms of juices or purees, and spices (ginger, cinnamon), among others.
3. Group 3: Smoothies A and B created the smallest group and were described by the terms viscosity, graininess, chalkiness, and aromatic. Smoothies were characterized by a high content of pineapple, orange, mango, carrot, pumpkin and ingredients unusual for other tested smoothies such as hemp seeds, citric fiber, and agave syrup, among others.
4. Group 4: This cluster was which included the most number of smoothies: C, D, F, H, J, and N. Consumers described them as familiar to them and probably, therefore, they used the largest number of attributes, including fruity, fresh fruity, overripe fruity, tropical fruit, and sweetness, among others. These products mainly contained banana, mango, grape, berries, orange, passion fruit, and peach, in crushed forms, purees and juices, as well as lemon and lime, among others.

For the F1 dimension, the highest % of contributions were bitterness, fruity, earthy, spicy, and beetroot variables (>4%), while, for the F2 dimension, citric, sourness and liquid were the highest contributors (>8%).

The terms sweetness, chalkiness, grainy, viscosity, dense, and aromatic were common to smoothies from Groups 3 and 4. Moreover, a clear differentiation among fruit smoothies and vegetable smoothies was found. The use of vegetable ingredients, such as carrot, beetroot, pumpkin, celery, cucumber, spinach, and kale, in a total amount of 31% to 33% for smoothies I, E, K, and M influenced the clearly perceptible vegetable, earthy, herbal, and astringency flavors, as well as spicy except for smoothie I. Terms for these vegetable smoothies were also associated with aftertaste, cooked notes, unknown and unpleasant flavors. As for cooked notes, it can be said that mild-heat treatment should have been applied during the smoothie processing as pasteurization (stored at refrigeration temperature), and could be the reason this flavor was detected by consumers.

Smoothies A and B were defined as dense, which could result not from the fruit and vegetable composition but from the presence of citric fiber with high water holding capacity and apparent viscosity. At this stage, the napping^®^ test showed no difference among sensory perception of smoothies with conventional and organic fruits and vegetables.

Finally, four smoothies representing each of these four clusters created by consumers in the Napping^®^ task were subjected to descriptive sensory analysis and consumer study (session 2) to obtained the improvements needed and the buying drivers: group 1: smoothie E, group 2: smoothie G, group 3: smoothie B, and group 4: smoothie D.

### 3.2. Descriptive Sensory Analysis

Napping^®^ itself does not characterize products; thus, there is a need of a descriptive method to get a full profile of the products [19]. Descriptive sensory analysis was carried out to assess the sensory profile of commercial smoothies and to check significant differences among the four groups; the main differences are shown in Table 4.

The study showed variability in the intensity of the main sensory descriptors. The sweetness of the smoothies was relatively low (maximum 4.5 points), and it can be observed that D samples was significantly higher than G and E samples. Smoothies B and G had the highest sour score (8.0 and 7.5, respectively). The presence of tropical fruits (mango, banana) was linked with higher scores of sweetness, while orange, pineapple, and berries with a higher intensity of sour taste. The results were in line with research on smoothies by other authors [29], where strawberries were linked with a sour flavor, and banana pulp with a high sweetness perception.

Product B, besides being sour, was characterized by the highest bitter notes and the longest aftertaste. Bitter notes were significantly lower in other smoothies and did not differ statistically (*p* > 0.05). A high value of aftertaste was also found in sample E, being both based on vegetable ingredients.

The detailed taste was determined based on descriptors of direct flavor of specific fruits and vegetables. Thus, the flavor of smoothie E with carrot (17%) and beet purees (14.5%) was defined as the highest scores of beetroot, earthy, and carrot (8.0, 9.0, and 6.0, respectively). The taste of this product was the most complex and described by seven attributes. Only this smoothie defined with low pear flavor notes. Smoothie B was described with only two flavor attributes, as orange and carrot, with the predominance of the first (scores 6.5 and 3.5, respectively).

In the smoothie G, only 7% aloe vera puree caused a medium intense flavor of this plant (score 5.0) and a slightly perceptible green-vinery hint. Additionally, low values of apple, orange, and citric flavors were detected. In product D, flavors in the decreasing intensity were mango > passion fruit > peach > apple and orange> banana were observed. The intensity of apple flavor reached up to 2.0, despite its 57% in the studied smoothies. Thus, apple seems to be a good base for creating and modulating flavor bouquets and as a carrier of ingredient flavors in fruit and vegetable smoothies without masking the minor ingredients. In other studies, apple improved the sensory acceptance of smoothies [15,18].

Commercial smoothies were not characterized by high values of the texture and somatic sensations attributes. Lumpiness was found only for smoothie B, and, in turn, tooth etch only for smoothie G. Pulpy residue and viscosity were higher in product B than the other smoothies. The evaluation of these attributes should consider not only the raw materials but also fineness/particle size (juice, puree, pulp, chopped pieces) and processing (cold, heat and enzymatic treatment).

### 3.3. Consumer Acceptability

#### 3.3.1. Hedonic Rating

Mean scores for liking of color, appearance, flavor notes (fruity, vegetal), basic tastes (sweetness, sourness), viscosity and overall liking of samples for a Spanish group of Millennial consumers are shown in Table 5.

In general, not all selected smoothies were considered acceptable, and can be ranked in decreasing order of overall liking D ≈ G > B > E, although smoothies D and G received the highest scores (6.4 and 5.6 points, respectively). For these products, the highest scores were found for fruity notes, sweetness, and sourness, which in turn were linked with the presence of mango, orange, banana, berries, passion fruit, and peach.

Clearly earthy flavors (e.g., beetroot and carrot) were not accepted by consumers. Therefore, smoothie E was rated overall very disliked (score 2.1), which resulted from low ratings for flavor notes and basic taste (below 4.5).

Color and appearance liking values were rated as the highest for smoothies E, B, and G. Fruit flavors were more liked than vegetables, and smoothie D was the best rated (score 7.0). However, no significant differences were found in the acceptance of vegetable flavors and bitterness (scores above 5.0), except for smoothie E (both attributes defined as dislike slightly). Sweetness and acidity were most liked in smoothie D. Furthermore, viscosity did not have a strong impact on the overall rating of the smoothies. This attribute was ranged between 2.4 (smoothie B previously defined as thick) to 6.3 (smoothies G).

Although previous studies examined consumer preferences for various fruits and vegetables for juices and smoothies, their results cannot be used directly as a reference to this paper. In this way, the sensory evaluation of smoothies was closely related to the set of products tasted, the profile of consumers, and their nutritional knowledge, economic and cultural factors, and others [30,31]. Literature data, however, indicates some recurring trends, which are also reflected in this study. Consumer preferences strongly correlate with the sensation of sweetness, intense fruit flavors (orange and tropical fruits), and balanced sweet and sour tastes [32,33,34].

#### 3.3.2. Penalty Analysis (PA)

**Figure 2:**
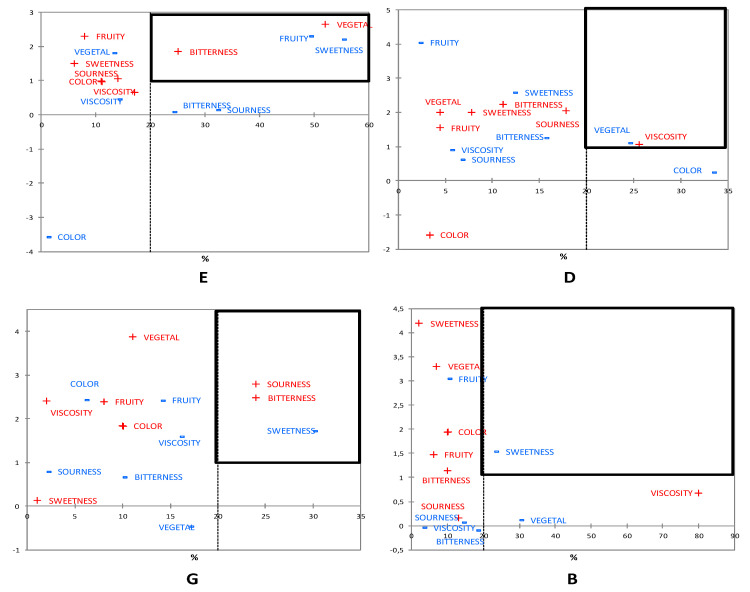
Penalty analysis of attribute intensity assessed by consumers of selected commercial smoothies (sample code indicated on the top right of each figure; “too low intensity” is indicated by the symbol “−”, and “too high intensity” is indicated by the symbol “+”).

In addition to the overall liking and the satisfaction degree for color, appearance, flavor notes, basic tastes, and viscosity, Just About Right (JAR) questions were asked during the consumer study among Spanish Millennial consumers under study, for all attributes except appearance and overall liking. The questions were done to determine levels of adequacy and identify possible improvements to these specific attributes in four selected smoothies. For better understanding of the relationships between JAR results and consumer acceptance, Penalty Analysis was conducted. “Too low” intensity of attributes was indicated by the symbol “−”, and “too high” intensity by the symbol “+” (Figure 2). Critical corners were set to highlight attributes with the greatest negative impact on liking. The attributes impacting 20% of Millennial consumers under study and causing a drop of at least one point were included in the critical corners. 

Specific food products are related to particular color attributes. Colors that diverge from these preferences for the product suggest a lack of ripeness or loss of freshness and may reduce consumer desire [34]. Smoothies were not penalized for the color. However, it was noted that smoothies with carrot, beet, pumpkin, and berries, containing high content of natural pigments (carotenoids and anthocyanins) were better rated in terms of color. The intensity of the fruity flavor needed improvement in product E, for which at the same time the vegetal taste was too intense. Interestingly, despite the low acceptance of vegetable smoothies, Millennial consumers under study rated the flavors of product D as not vegetal enough. Smoothies, except for D, need a sweetness improvement. In turn, the sour taste should be corrected in smoothie G. The blend of orange and berries caused too intense feelings of sourness and bitterness, in contrast to root vegetable combinations. Too high viscosity was determined for smoothie D. In summary, the color intensity and perception of the fruity taste were the best rated. It is essential to highlight that fruity notes were clearly driving liking as in the following section. This tendency does not occur in all fruit-based products. For instance, in pomegranate-based jelly, fruit notes were not a good driver, with color and brightness being the most important drivers [21], but, in pomegranate dried products, both fruity and color attributes were part of the consumer drivers [23]. Four of the seven sensory attributes were found to be improvable in smoothie E, a model of the group 1 cluster.

### 3.4. Driving Sensory Attributes

**Figure 3:**
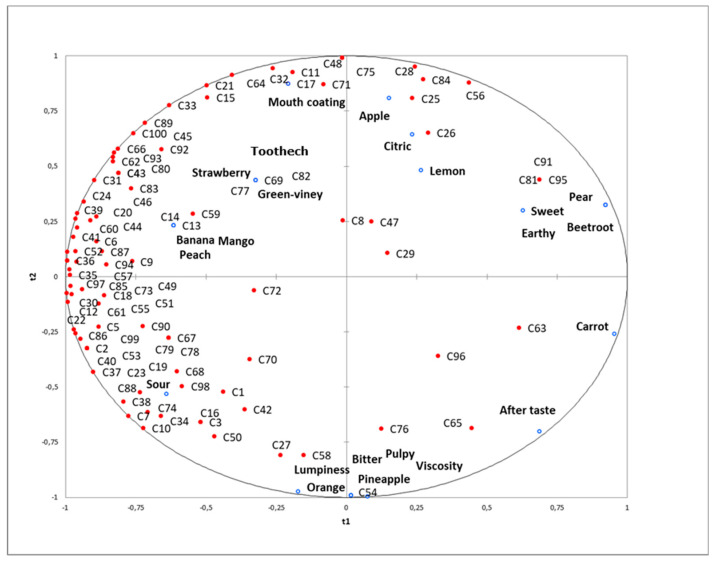
Partial least squares regression (PLS) of the descriptive sensory profile (Y) and consumer overall liking (X) of selected commercial smoothies.

Partial least squares regression (PLS) analysis was conducted to determine the liking drivers for the selected commercial smoothies. Figure 3 shows the consumer overall liking and the relationship with specific sensory descriptors (contained in Table 3).

The preference map revealed one large group of consumers grouped near mango, peach, and banana flavors. Similar results were obtained previously [35,36,37] in the analysis of smoothies, where blends of papaya, mango, and pineapple as well as mango and grape were highly desired by panelists. However, contrary to their results, a combination of banana with fruits with high acidity were clearly accepted by Millennial consumers.

The current study showed that the lowest acceptance of smoothies was associated with earthy, carrot, beetroot, and pear flavors.

Considering the basic tastes, sour was the main liking driver, while sweet, bitter, and aftertaste seemed to be less important. These results were in line with the penalty analysis data, in which sweetness and bitterness required correction. Interestingly, previous studies on fresh fruits, including pineapples [37], strawberries [38], nectarines and peaches [32], and apples [30,39] showed sweetness as a positive attribute, often even as the main sensory preference driver. Other research into fruit products such as oranges juice [33], pear fruit leather [40], dried pomegranate arils [23], as well as jelly candies from pomegranate juice [21], indicated a strong correlation between general acceptability and sensation of sweetness. However, another study reported sourness as a key apple acceptance driver [39], although it depends on the apple cultivar (sour, and sweet ones).

Lumpiness, viscosity, and pulpy were not appreciated by Millennial consumers under study. Forde and Delahunty [31] proved that young panelists can be strongly influenced by the predominant chemosensory attributes such as taste and aroma level [31], while older assessors placed more emphasis on the mouthfeel and irritant properties. Moreover, the homogeneity of the sample is preferred by younger consumers as compared to older ones; this situation was observed in the current study. Therefore, it should be emphasized that the main liking drivers for consumers of smoothies are sourness and tropical fruit flavors, and earthy and heterogeneous textures must be avoided.

## 4. Conclusions

This research evaluated sensory properties of commercial smoothies and identified liking drivers among the Spanish group of Millennial consumers under study. At the beginning of this conclusion, it is worth mentioning the limitation of this research to help and to make future research better. The sample size of consumers is one of the most important limitations observed, and the number of consumers should be higher to reach the global Millennial generation. It is also important to highlight the potential interactions based on the flavor and basic tastes that can occur; therefore, samples with specific flavor (e.g., mango) could be scored sweetener by consumers (never by trained panel) due to their expectations.

Descriptive sensory analysis and consumer studies preceded by the napping^®^ test seem to be an appropriate combination to optimize the formulation of novel fruit- and vegetable-smoothies. It was found that the key attributes controlling overall liking were: adequate intensity of sour taste and notes of mango, banana, and peach. Nevertheless, it should strive to improve recipes of smoothies to increase the consumption of fruits and vegetables in this form, which is considered a simple supplement in a balanced diet. Results of the penalty analysis can give a good direction to optimize this type of smoothies by avoiding vegetable ingredients with earthy or strong vegetal notes (e.g., beetroot, carrot). Research provides a series of practical tips for the food industry to understand consumer preferences, select raw materials, and improve marketing strategies. Knowing the separate clusters of smoothies available on the market and the drivers of their preferences, highly acceptable products can be developed targeting a specific consumer profile.

## Figures and Tables

**Table 1:** Composition of commercial smoothies used in this study.

Ingredient	Smoothie (%)													
	A	B	C	D	E	F	G	H	I	J	K	L	M	N
**Fruit Purée**														
Apple	†	†	0	0	0	3 ½ ^‡^	0	1 ½ ^‡^	0	1 ½ ^‡^	0	0	0	0
Apple puree	0	0	0	0	0	0	0	0	11	0	0	0	22	0
Apple sauce	0	0	0	0	21	0	0	0	0	0	0	0	0	0
Banana	†	0	0	0	0	1 ½ ^‡^	0	0	0	2 ^‡^	0	0	0	0
Banana puree	0	0	20	15	0	0	0	¾ ^‡^	0	0	0	5	0	10
Blackberry puree	0	0	8	0	0	0	0	0	0	0	0		0	0
Blackcurrant	0	0	0	0	0	0	0	9 ^‡^	0	17 ^‡^	0	0	0	0
Blueberry puree	0	0	0	0	0	0	6	0	0	0	0	5	0	0
Cherries	0	0	0	0	0	0	0	4 ^‡^	0	0	0	0	0	0
Currant puree	0	0	2	0	0	0	0	0	0	0	0	0	0	0
Guarana	0	0	0	0	0	0	0	†	0	0	0	0	0	0
Kiwi	†	0	0	0	0	0	0	0	0	0	0	0	0	0
Lime	0	0	0	0	0	†	0	0	0	0	0	0	0	0
Mango	7.7	0	0	0	0	17	0	0	0	0	0	0	0	0
Mango puree	0	0	0	16	0	0	0	0	0	0	0	0	0	15
Orange pulp	0	0	6	0	0	0	0	0	0	0	0	0	0	0
Passion fruit	0	0.2	0	0	0	¼ ^‡^	0	0	0	0	0	0	0	0
Peach puree	0	0	0	2	0	†	0	0	0	0	0	0	0	0
Pear puree	0	0	0	0	0	0	0	0	0	0	17	0	0	0
Pineapple	10.5	11	0	0	0	0	0	0	0	0	0	0	0	0
Raspberry puree	0	0	8	0	0	0	6	0	0	0	0	17	0	0
Red grapes	0	0	0	0	0	0	0	14 ^‡^	0	0	0	0	0	0
Strawberry	0	0	0	0	0	0	0	0	0	22 ^‡^	0	0	0	0
Strawberry puree	0	0	9	0	0	0	4	3 ½ ^‡^	0	0	0	20	0	0
White grape	0	0	0	0	0	0	0	0	0	41 ^‡^	0	0	0	0
**Fruit and Vegetable Juices/Concentrated**														
Apple juice	0	0	39	52	43	0	57	0	41	0	0	53	43	71.5
Apple juice from concentrate	†	0	0	0	0	0	0	0	0	0	0	0	0	0
Beet juice	0	0	0	0	0	0	0	†	0	0	0	0	0	0
Cucumber juice	0	0	0	0	0	0	0	0	0	0	10	0	0	0
Ginger juice	0	0	0	0	0	0	0	0	0	0	0	0	1	0
Grape juice	0	0	10	0	0	0	0	0	0	0	0	0	0	0
Lemon juice	0	0	0	†	4	0	0	0	6	0	4.5	0	2	0
Orange juice	0	39.5	0	10	0	1^‡^	15	0	0	¼ ^‡^	0	0	0	0
Passion fruit juice	0	0	0	4	0	0	0	0	0	0	0	0	0	3.5
Pear juice	0	0	0	0	0	0	0	0	0	0	28	0	0	0
Pineapple juice	24	0	0	0	0	0	0	0	10	0	18	0	0	0
Pomegranate juice	0	0	0	0	0	0	5	0	0	0	0	0	0	0
Romaine lettuce juice	0	0	0	0	0	0	0	0	0	0	3.5	0	0	0
**Vegetables Purée**														
Aloe vera puree	0	0	0	0	0	0	7	0	0	0	0	0	0	0
Beet puree	0	0	0	0	14.5	0	0	0	0	0	0	0	0	0
Carrot	†	15	0	0	0	0	0	0	0	0	0	0	0	0
Carrot puree	0	0	0	0	17	0	0	0	17	0	0	0	0	0
Celery	0	0	0	0	0	0	0	0	0	0	13	0	0	0
Celery puree	0	0	0	0	0	0	0	0	0	0	0	0	8	0
Cucumber puree	0	0	0	0	0	0	0	0	0	0	0	0	14	0
Iceberg lettuce	6	0	0	0	0	0	0	0	0	0	0	0	0	0
Kale puree	0	0	0	0	0	0	0	0	0	0	0	0	5	0
Ginger puree	0	0	0	0	<0.1	0	0	0	0.1	0	0	0	0	0
Sweet corn	†	0	0	0	0	0	0	0	0	0	0	0	0	0
Pumpkin	0	7	0	0	0	0	0	0	0	0	0	0	0	0
Pumpkin puree	0	0	0	0	0	0	0	0	14	0	0	0	0	0
Spinach puree	0	0	0	0	0	0	0	0	0	0	5	0	5	0
**Others**														
Agave syrup	0	†	0	0	0	0	0	0	0	0	0	0	0	0
Ascorbic acid	0	0	0	0	0	0	†	0	0	0	†	0	0	0
Cinnamon	0	0	0	0	0	0	0	0	<0.1	0	0	0	0	0
Citric fiber	†	†	0	0	0	0	0	0	0	0	0	0	0	0
Coconut milk	4	0	0	0	0	0	0	0	0	0	0	0	0	0
Ground flax seeds	0	0	0	0	0	0	0	†	0	0	0	0	0	0
Hemp seeds	0.8	0	0	0	0	0	0	0	0	0	0	0	0	0
Natural aroma	†	0	0	0	0	0	0	0	0	0	0	0	0	0
Nettles	0	0	0	0	0	0	0	0	0	0	†	0	0	0
Vitamin infusion	0	0	0	0	0	0	0	†	0	0	0	0	0	0

† Presence but quantity was not indicated on ingredient list. ‡ ingredient expressed as fruit pieces and not as %.

**Table 2:** Lexicon of the sensory descriptors.

Attribute	Definition	References	Previous Publications
After taste	Longevity of key attributes intensity after swallow the sample	5 s = 1.0; 30 s = 4.0; 60 s = 8.0	[17,22]
Bitter	The basic taste associated with a caffeine solution	0.008% caffeine solution = 1.0 0.15% citric acid solution = 2.0	[17,22,26]
Sour	The taste factor associated with some organic acid, specifically citric acid	0.043% citric acid solution = 2.0 0.064% citric acid solution = 3.0 0.120% citric acid solution = 5.0 0.168% citric acid solution = 7.0	[17,22]
Sweet	The basic taste associated with a sucrose solution	3% sucrose solution = 2.0 6% sucrose solution = 4.0 12% sucrose solution = 8.0	[17,22]
Aloe	Aromatic associated with aloe	Diluted aloe juice (1:1) = 5 Fresh aloe juice = 10	[26]
Apple	Aromatic compounds associated with processed apple juice and cooked apples	Hacendado mango–apple nectar = 5.5	[17]
Banana	Aromatic associated with bananas	Fresh peeled banana = 10	[26]
Beetroot	The damp, musty/earthy, slightly sweet aromatics commonly associated with canned/cooked beets	Diluted kroger canned beet juice (1:2) = 4.0	[24]
Carrot	The aromatics commonly associated with canned, cooked carrots	Del monte sliced canned carrots = 7.0	[26]
Citric	Volatile compounds associated with lemon or lime	Fresh-squeezed orange juice = 8 Fresh-squeezed orange juice diluted 1:1 = 4	[22]
Earthy	Musty, somewhat sweet, full aromatics commonly associated with decaying vegetative matter and damp black soil	Geosmin (4,000 ppm) = 9.0	[27]
Green-vinery	Green, fresh aromatics associated with green vegetables and newly cut vines and stems; related to cucumber	Trans-2-hexen-1-ol 5000 ppm = 4.0 Heinz tomato ketchup (vinegar) = 4.5 Freshly sliced tomatoes = 10.0	[24]
Mango	A sweet, fruity aromatic associated with mango	Fresh peeled mango = 10	[26]
Orange	The aromatics associated with oranges; including juice, pulp and peel	Fresh-squeezed orange juice = 9	[26]
Passion fruit	A sweet, fruity aromatic associated with passionfruit	Fresh passionfruit = 10	[26]
Peach	Aromatic compounds from ripe peach	Fresh peeled peach = 10	[26]
Pear	Sweet, slightly musty, floral, honey/caramel-like, fruity aromatic associated with ripe pears	Hacendado pear nectar = 6.5	[17]
Lumpiness	The perception of large particles that are not dissolved in the product	Yoplait Strawberry Yogurt = 4.0	
Mouth coating	The amount of film left on the mouth surfaces	Whipped cream = 6 Pureed potato = 10	
Pulpy	A soft moist residue	Del Monte slices peaches = 2	
Tooth etch	A sensation of abrasion and drying of the surface of the teeth.	Welch’s grape juice diluted (1:1) = 6.0	[24,25]
Viscosity	The force required to move the product across the tongue	Distilled water = 1 Condensed milk diluted (1:1) = 5 Condensed milk =10	

**Table 3:** Terms used by Millennial consumers to describe commercial smoothies in the Napping^®^ test and number of mentions for each of the samples, considering that 100 consumers participated in the test.

Term	Smoothie														Number of Term Repetitions
	A	B	C	D	E	F	G	H	I	J	K	L	M	N	
Fruity	16	17	30	32	6	34	16	22	22	26	0	23	1	28	273
Fresh Fruity	9	9	11	16	3	20	18	19	18	16	2	19	2	11	173
Overripe fruity	16	12	16	19	6	19	10	15	7	19	1	9	1	20	170
Citric	5	26	21	15	1	14	43	19	52	16	0	46	0	23	281
Berries	1	3	11	1	1	0	7	9	3	10	0	9	0	0	55
Tropical fruit	18	17	28	39	3	38	22	30	19	29	0	22	0	33	298
Stone fruit	3	7	5	12	1	12	3	5	2	5	0	0	1	6	62
Caramel	0	2	4	3	2	4	1	6	2	6	0	2	0	4	36
Cooked notes	6	5	3	2	10	1	4	5	0	3	9	1	8	2	59
Earthy	16	8	4	1	24	0	1	1	3	1	26	0	22	2	109
Herbal	15	3	2	2	29	1	2	1	3	2	41	2	41	2	146
Vegetal	16	8	3	2	53	2	8	2	6	2	61	0	59	5	227
Spicy	7	4	2	2	10	1	4	2	0	2	14	2	14	3	67
Beetroot	3	0	0	1	21	1	5	1	4	2	19	3	19	0	79
Familiar	8	8	9	21	3	19	9	13	10	19	3	14	3	17	156
Unknown	5	2	2	0	5	0	1	1	4	1	8	0	7	0	36
Apple	4	4	12	6	1	6	2	12	2	9	0	6	0	1	65
Vegetable	16	5	2	1	41	0	4	2	10	1	48	1	47	3	181
After-taste	16	11	10	8	11	8	13	10	16	6	19	13	17	13	171
Artificial	2	2	2	1	2	0	1	1	1	0	4	0	4	3	23
Sweetness	30	16	29	63	10	59	13	34	14	44	1	18	2	50	383
Sourness	6	19	32	15	3	15	48	27	53	21	2	57	2	22	322
Bitterness	8	12	7	3	23	2	12	7	8	2	28	8	30	3	153
Astringency	3	7	5	0	3	1	9	7	8	4	4	8	5	2	66
Chalkiness	9	17	13	4	8	7	7	12	6	11	8	5	8	6	121
Grainy	22	27	15	9	3	8	7	10	8	18	9	9	5	10	160
Viscosity	25	25	17	11	6	10	8	15	10	18	5	10	5	9	174
Liquid	1	7	8	11	10	13	22	11	21	5	7	19	6	17	158
Persistent	1	1	1	1	1	0	0	0	1	1	2	1	1	0	11
Flat	4	6	3	2	6	1	2	1	2	2	2	1	4	4	40
Unpleasant	2	1	0	0	4	0	1	0	0	0	7	0	7	1	23
Dense	8	6	4	3	1	4	0	3	4	3	2	1	1	2	42
Aromatic	2	1	0	1	1	1	0	1	0	0	0	0	0	2	9
Soft	1	0	2	1	1	2	1	3	0	2	0	1	0	2	16
Number of Terms of Each Sample	304	298	313	308	313	303	304	307	319	306	332	310	322	306	

**Table 4:** Descriptive sensory analysis of the selected commercial smoothies. The scale was range from 0 (no intensity) to 10 (extremely high intensity) with 0.5 increments.

Attribute	ANOVA ^†^	Smoothie			
		E	B	G	D
Basic taste					
After taste	**	7.0 ^‡^ ab	7.5 a	6.0 b	5.5 b
Bitter	*	0.5 b	1.5 a	0.5 b	0.5 b
Sour	***	3.0 c	8.0 a	7.5 a	5.5 b
Sweet	*	2.5 b	3.5 ab	3.0 b	4.5 a
Flavor					
Aloe	***	0.0 b	0.0 b	5.0 a	0.0 b
Apple	**	2.0 a	0.0 b	1.0 ab	2.0 a
Banana	*	0.0 b	0.0 b	0.0 b	1.5 a
Beetroot	***	8.0 a	0.0 b	0.0 b	0.0 b
Carrot	***	6.0 a	3.5 b	0.0 c	0.0 c
Citric	***	1.5 b	0.0 c	2.5 a	0.0 c
Earthy	***	9.0 a	0.0 b	0.0 b	0.0 b
Green-vinery	*	0.0 b	0.0 b	2.0 a	0.0 b
Mango	***	0.0 b	0.0 b	0.0 b	5.0 a
Orange	***	1.0 b	6.5 a	2.0 b	2.0 b
Passion fruit	***	0.0 b	0.0 b	0.0 b	4.0 a
Peach	**	0.0 b	0.0 b	0.0 b	3.5 a
Pear	*	1.5 a	0.0 b	0.0 b	0.0 b
Texture and somatic sensations					
Lumpiness	***	0.0 b	5.5 a	0.0 b	0.0 b
Mouth coating	*	1.0 ab	0.5 b	1.5 a	1.0 ab
Pulpy	**	0.0 b	3.5 a	0.0 b	0.0 b
Tooth etch	***	0.0 b	0.0 b	4.5 a	0.0 b
Viscosity	***	1.5 b	4.0 a	1.0 b	1.5 b

^†^ *, **, and *** significant at *p* < 0.05, 0.01, and 0.001, respectively. ^‡^ Values (mean of eight trained panelists) followed by the same letter within the same row were not significantly different according to Tukey’s least significant difference test. Scale used ranged from 0 (no intensity) to 10 (extremely strong intensity).

**Table 5:** Mean scores and ANOVA for liking degree of a Spanish group of Millennial consumers for color, appearance, flavor notes (fruity, vegetal), basic tastes (sweetness, sourness), viscosity and overall liking of selected commercial smoothies.

Attribute	ANOVA ^†^	Smoothie’s			
		E	B	G	D
Appearance	***	6.8 ^‡^ a	6.5 a	6.1 a	4.8 b
Color	***	7.6 a	7.3 a	6.3 b	5.0 c
Bitterness	***	4.2 b	5.2 a	5.1 a	5.9 a
Sourness	***	4.5 c	5.4 b	5.6 ab	6.2 a
Sweetness	***	4.1 c	5.7 ab	5.5 b	6.5 a
Fruity	***	3.7 c	6.0 b	6.1 b	7.0 a
Vegetal	***	3.8 b	5.9 a	5.8 a	6.3 a
Viscosity	***	5.6 ab	2.4 c	6.3 a	5.3 b
Overall liking	***	2.1 c	4.8 b	5.6 a	6.4 a

^†^ *** significant at *p* < 0.001. ^‡^ Values (mean of 100 consumers) followed by the same letter within the same row were not significantly different according to Tukey’s least significant difference test. Scale used from 1 (dislike it extremely) to 9 (like it extremely).

## References

[1] González-Tejedor G.A., Martínez-Hernández G.B., Garre A., Egea J.A., Fernández P.S., Artés-Hernández F. (2017). Quality changes and shelf-life prediction of a fresh fruit and vegetable purple smoothie. Food Bioprocess Technol..

[2] Trichopoulou A., Vasilopoulou E., Caballero B., Finglas P.M., Toldrá F. (2016). Mediterranean diet. Encyclopedia of Food and Health.

[3] Murphy M.M., Barrett E.C., Bresnahan K.A., Barraj L.M. (2017). 100% Fruit juice and measures of glucose control and insulin sensitivity: A systematic review and meta-analysis of randomised controlled trials. J. Nutr. Sci..

[4] Hall J.N., Moore S., Harper S.B., Lynch J.W. (2009). Global variability in fruit and vegetable consumption. Am. J. Prev. Med..

[5] Castillejo Montoya N., Martínez-Hernández G.B., di Marco G., Azucena P., Artés Calero F., Artés Hernández F.D.A. (2015). Red fresh vegetables smoothies with extended shelf life as an innovative source of health-promoting compounds. J. Food Sci. Technol. Mysore.

[6] MAPA (2018). Informe Del Consumo Alimentario en España 2018.

[7] Smith V., Green-Petersen D., Møgelvang-Hansen P., Christensen R.H.B., Qvistgaard F., Hyldig G. (2013). What’s (in) a real smoothie. A division of linguistic labour in consumers′ acceptance of name–product combinations?. Appetite.

[8] Stan A., Popa M. (2013). Research on the correlation between physico-chemical, sensory analysis of smoothie type products and consumer preferences. Sci. Bull. Ser. F Biotechnol..

[9] Market Data Forecast: Smoothies Market Analysis by Product (Fruit-Based, Dairy-Based and Others), by Distribution Channel (Restaurants, Smoothie Bars, Supermarkets and Convenience Stores) and by Region (North America, Europe, Asia Pacific, Latin America, and Middle East and Africa)—Global Industry Size, Share, Growth, Trends, Demand and Forecast Report 2020–2025. https://www.marketdataforecast.com/market-reports/smoothies-market.

[10] AIJN European Fruit Juice Association (2016). Liquid Fruit Market Report.

[11] Mastrolia S., Willits S. (2013). Millennials: What do we really know about them?. Advances in Accounting Education: Teaching and Curriculum Innovations.

[12] Andrés V., Villanueva M.-J., Tenorio M.-D. (2016). Influence of high pressure processing on microbial shelf life, sensory profile, soluble sugars, organic acids, and mineral content of milk-and soy-smoothies. LWT Food Sci. Technol..

[13] Di Cagno R., Minervini G., Rizzello C.G., De Angelis M., Gobbetti M. (2011). Effect of lactic acid fermentation on antioxidant, texture, color and sensory properties of red and green smoothies. Food Microbiol..

[14] Keenan D.F., Brunton N.P., Mitchell M., Gormley R., Butler F. (2012). Flavour profiling of fresh and processed fruit smoothies by instrumental and sensory analysis. Food Res. Int..

[15] Nowicka P., Wojdyło A., Teleszko M., Samoticha J. (2016). Sensory attributes and changes of physicochemical properties during storage of smoothies prepared from selected fruit. LWT Food Sci. Technol..

[16] Walkling-Ribeiro M., Noci F., Cronin D.A., Lyng J.G., Morgan D.J. (2010). Shelf life and sensory attributes of a fruit smoothie-type beverage processed with moderate heat and pulsed electric fields. LWT Food Sci. Technol..

[17] Issa-Issa H., Cano-Lamadrid M., Calín-Sánchez Á., Wojdyło A., Carbonell-Barrachina Á.A. (2020). Volatile composition and sensory attributes of smoothies based on pomegranate juice and mediterranean fruit purées (fig, jujube and quince). Foods.

[18] Teleszko M., Wojdyło A. (2014). Bioactive compounds vs. organoleptic assessment of ‘smoothies’-type products prepared from selected fruit species. Int. J. Food Sci. Technol..

[19] Macfie H.J., Bratchell N., Greenhoff K., Vallis L.V. (1989). Designs to balance the effect of order of presentation and first-order carry-over effects in hall tests. J. Sens. Stud..

[20] Perrin L., Symoneaux R., Maître I., Asselin C., Jourjon F., Pagès J. (2008). Comparison of three sensory methods for use with the Napping^®^ procedure: Case of ten wines from Loire valley. Food Qual. Prefer..

[21] Cano-Lamadrid M., Calín-Sánchez Á., Clemente-Villalba J., Hernández F., Carbonell-Barrachina Á.A., Sendra E., Wojdyło A. (2020). Quality parameters and consumer acceptance of jelly candies based on pomegranate juice “Mollar de Elche”. Foods.

[22] Cano-Lamadrid M., Lipan L., Hernández F., Martínez J.J., Legua P., Carbonell-Barrachina Á.A., Melgarejo P. (2018). Quality parameters, volatile composition, and sensory profiles of highly endangered Spanish citrus fruits. J. Food Qual..

[23] Cano-Lamadrid M., Vázquez-Araújo L., Sánchez-Rodríguez L., Wojdyło A., Carbonell-Barrachina Á.A. (2018). Consumers′ opinion on dried pomegranate arils to determine the best processing conditions. J. Food Sci..

[24] Koppel K., Chambers E. (2010). Development and application of a lexicon to describe the flavor of pomegranate juice. J. Sens. Stud..

[25] Hongsoongnern P., Chambers E. (2008). A lexicon for texture and flavor characteristics of fresh and processed tomatoes. J. Sens. Stud..

[26] Meilgaard M.C., Thomas Car B., Vance-Civille G. (2006). Sensory Evaluation Techniques.

[27] Talavera-Bianchi M., Chambers E., Chambers D.H. (2010). Lexicon to describe flavor of fresh leafy vegetables. J. Sens. Stud..

[28] Pathare P.B., Opara U.L., Al-Said F.A.-J. (2013). Colour measurement and analysis in fresh and processed foods: A review. Food Bioprocess Technol..

[29] De Oliveira Ribeiro L., Carvalho Dos Santos J.G., dos Santos Gomes F., Correa Cabral L.M., de Grandi Castro Freitas SÁ D., Martins da Matta V., Freitas S.P. (2017). Sensory evaluation and antioxidant capacity as quality parameters in the development of a banana, strawberry and juçara smoothie. Food Sci. Technol..

[30] Endrizzi I., Torri L., Corollaro M.L., Demattè M.L., Aprea E., Charles M., Biasioli F., Gasperi F. (2015). A conjoint study on apple acceptability: Sensory characteristics and nutritional information. Food Qual. Prefer..

[31] Forde C., Delahunty C. (2004). Understanding the role cross-modal sensory interactions play in food acceptability in younger and older consumers. Food Qual. Prefer..

[32] Delgado C., Crisosto G.M., Heymann H., Crisosto C.H. (2013). Determining the primary drivers of liking to predict consumers′ acceptance of fresh nectarines and peaches. J. Food Sci..

[33] Kim M.K., Lee Y.J., Kwak H.S., Kang M.W. (2013). Identification of sensory attributes that drive consumer liking of commercial orange juice products in Korea. J. Food Sci..

[34] Barrett D.M., Beaulieu J.C., Shewfelt R. (2010). Color, flavor, texture, and nutritional quality of fresh-cut fruits and vegetables: Desirable levels, instrumental and sensory measurement, and the effects of processing. Crit. Rev. Food Sci. Nutr..

[35] Balaswamy K., Rao P.P., Nagender A., Rao N.G., Mala S.K., Jyothirmayi T., Math R., Satyanarayana A. (2013). Development of smoothies from selected fruit pulps/juices. Int. Food Res. J..

[36] Jan A., Masih E.D. (2012). Development and quality evaluation of pineapple juice blend with carrot and orange juice. Int. J. Sci. Res. Publ..

[37] Schulbach K.F., Portier K.M., Sims C.A. (2007). Evaluation of overall acceptability of fresh pineapple using the regression tree approach. J. Food Qual..

[38] Lado J., Vicente E., Manzzioni A., Ares G. (2010). Application of a check-all-that-apply question for the evaluation of strawberry cultivars from a breeding program. J. Sci. Food Agric..

[39] Symoneaux R., Galmarini M., Mehinagic E. (2012). Comment analysis of consumer’s likes and dislikes as an alternative tool to preference mapping. A case study on apples. Food Qual. Prefer..

[40] Huang X., Hsieh F.H. (2005). Physical properties, sensory attributes, and consumer preference of pear fruit leather. J. Food Sci..

